# Supplementary material for: PagbHLH35 Enhances Salt Tolerance through Improving ROS Scavenging in Transgenic Poplar
Source: Plants (Basel). 2024 Jul 3;13(13):1835. doi: 10.3390/plants13131835 (PMC11244237; doi:10.3390/plants13131835)
Supplement: Supplementary file 1 [file plants-13-01835-s001.zip › Table S1 Primer sequences used in the analysis of PagbHLH35.pdf]

Supplementary Table S1. Primer sequences used in the analysis of *PagbHLH35*

Primer	Sequence (5'-3')
PagbHLH35-F	ATGGAATCTTTTCAGAACATTTCTG
PagbHLH35-R	CTACATGCCTTGAGGGTTATATGG
DL- bHLH35-F	ATCAACTTAGGGTCTCGTCCAT
DL- bHLH35-R	GTGTCTTCTTGACCATCCCTGA
PagActin-F	TCAACCGCCTTGTCTCTCAGG
PagActin-R	TGGCTCGAATGCACTGTTGG
pBI121-bHLH35-F	GCTCTAGAATGGAATCTTTTCAGAACATTTCTG
pBI121-bHLH35-R	GGACTAGTCATGCCTTGAGGGTTATATGG
pBI121-F	CAACCACGTCTTCAAAGCAAG
pBI121-R	TCGAGCTCCTACTTGTCGTCA
pGBKT7-bHLH35-F	GGAATTCATGGAATCTTTTCAGAACATTTCTG
pGBKT7-bHLH35-R	CGGGATCCCATGCCTTGAGGGTTATATGG
pGADT7-BHLH35-F	CCCGGGATGGAATCTTTTCAGAACATTTCTG
pGADT7-BHLH35-R	CTCGAGCTACATGCCTTGAGGGTTATATGG
E-box-F	AGCTCCAAATGCAAATGCAAATGC
E-box-R	GCATTTGCATTTGCATTTGGAGCT
G-box-F	AGCTCCACGTGCACGTGCACGTGC
G-box-R	GCACGTGCACGTGCACGTGGAGCT
PabAi-F	CATGATTTATCTTCGTTTCCTGC
PabAi-R	CAGTTTGGAGGTCTCTCTGATAGA
DL- bHLH35-F	ATCAACTTAGGGTCTCGTCCAT
DL- bHLH35-R	GTGTCTTCTTGACCATCCCTGA
POD1-F	GCTGAGTCCATTGTTAGATC
POD1-R	GCCTGCCTCTCAATGGAACG
POD2-F	GTTCCATCTCTTACTTGTTC
POD2-R	CTTGTTGGTTGCAGTGGAGG
SOD1-F	GCCTTGCCTGAGATACTTAC
SOD1-R	GCTTCAGTCATAGTCTTCAC
SOD2-F	ACGCATCCATTTGTTGTGTC
SOD2-R	ACGCATCCATTTGTTGTGTC
