# Supplementary material for: PagbHLH35 Enhances Salt Tolerance through Improving ROS Scavenging in Transgenic Poplar
Source: Plants (Basel). 2024 Jul 3;13(13):1835. doi: 10.3390/plants13131835 (PMC11244237; doi:10.3390/plants13131835)
Supplement: Supplementary file 1 [file plants-13-01835-s001.zip › Table S2 Specific motif sequences of BHLH transcription factor.pdf]

Supplementary Table S2 Specific motif sequences of *BHLH* transcription factor.

Primer	Sequence (5'-3')
Motif 1	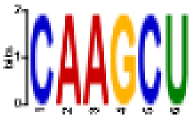
Motif 2	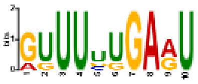
Motif 3	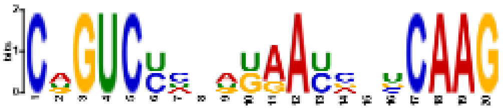
Motif 4	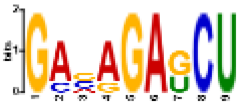
Motif 5	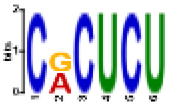
Motif 6	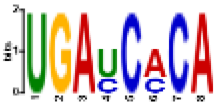
Motif 7	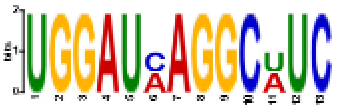
Motif 8	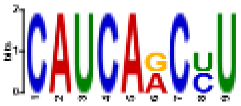
Motif 9	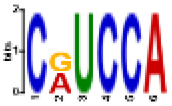
Motif 10	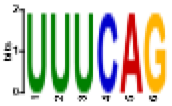
